# Utility of SAM68 in the progression and prognosis for bladder cancer

**DOI:** 10.1186/s12885-015-1367-x

**Published:** 2015-05-06

**Authors:** Zhiling Zhang, Chunping Yu, Yonghong Li, Lijuan Jiang, Fangjian Zhou

**Affiliations:** 1 State Key Laboratory of Oncology in Southern China, Guangzhou, China; 2 Department of Urology, Sun Yat-sen University Cancer Center, Guangzhou, China; 3 Collaborative Innovation Center for Cancer Medicine, Guangzhou, China; 4 Department of Nephrology, Guangdong General Hospital, Guangdong Academy of Medical Sciences, Guangzhou, China

**Keywords:** SAM68, Bladder cancer, Prognosis, Biomarker, Progression

## Abstract

**Background:**

Muscle invasive bladder cancer (MIBC) is often lethal and non-MIBC (NMIBC) can recur and progress, yet prognostic markers are currently inadequate. SAM68, a member of RNA-binding proteins, has been reported to contribute to progression of other cancers. The aim of this study is to investigate the potential utility of SAM68 in the progression and prognosis of bladder cancer.

**Methods:**

Quantitative PCR and immunohistochemistry were utilized to examine the expression of SAM68 in ten pairs of MIBC and adjacent normal bladder urothelium, and eight pairs of MIBC and non-MIBC (NMIBC) tissues from the same patient. Moreover, SAM68 protein expression level and localization were examined by immunohistochemistry in 129 clinicopathologically characterized MIBC samples. Prognostic associations were determined by multivariable analysis incorporating standard prognostic factors.

**Results:**

SAM68 expression was elevated in MIBC tissues compared with adjacent normal bladder urothelium, and was increased at both transcriptional and translational levels in MIBC tissues compared with NMIBC tissues of the same patient. For MIBC, high expression and nucleus-cytoplasm co-expression of SAM68 were associated with higher T-stage, higher N-stage and worse recurrence-free survival. Five-year recurrence-free survival was 80% and 52.9% for MIBC patients with low and high SAM68 expression, respectively (p = 0.001). SAM68 nucleus-cytoplasm co-expression associated with worse 5-year recurrence-free survival rate (49.2%) than SAM68 expression confined to the nucleus (82.5%) or cytoplasm (75.5%) alone. On multivariable analysis SAM68 expression level, SAM68 nucleus-cytoplasm co-expression, T-stage, and N-stage were all independent prognostic factors for recurrence-free survival of MIBC patients.

**Conclusions:**

SAM68 expression is increased in MIBC when compared to normal urothelium and NMIBC, and appears to be a potentially useful prognostic marker for MIBC.

## Background

Bladder cancer is the fourth most-common malignancy in men, accounting for 7% of all cancer cases. In 2014 there will be 74,690 new cases of bladder cancer in the USA alone, leading to 15,580 cancer-related deaths [1]. About 25% of bladder cancers are muscle invasive (MIBC) at presentation [2,3], and such patients are at high risk of cancer death even after intensive treatment, with a five-year survival rate of 33% and 5.4% for regional and distal metastasis MIBC, respectively [4]. Approximately 1/3 patients have cancer recurrence after cystectomy even with adjuvant chemotherapy [5]. Although altered expression of oncogenes, tumor suppressors, and other markers have been found in bladder cancer [6], the prognostic utility of currently available biomarkers remains limited, and they are not routinely incorporated into clinical practice. The identification of novel biomarkers involved in the progression of bladder cancer would greatly facilitate patient management.

SAM68 (Src-associated in mitosis, 68 kDa) is a member of the STAR (signal transduction and activation of RNA) family of RNA-binding proteins [7], and has shown potential as a biomarker for malignancy [8-11]. SAM68 proteins have an hnRNP K homology domain (KH domain) that locates within a larger GSG (GRP33-SAM68-GLD1) domain that is required for specificity and high-affinity binding to RNA [7,12]. This multimodular structure allows SAM68 protein to exert different functions in the cell, including regulation of the cell cycle, proliferation, and apoptosis. SAM68 has been reported to associate with progression and/or prognosis for a variety of malignancies, including renal cell carcinoma [8], prostate cancer [9], cervical cancer [10] and breast cancer [11]. For example, increased expression and cytoplasmic localization of SAM68 correlated with clinical outcomes and prognosis for patients with breast cancer. An *in vitro* study revealed that down-regulation of SAM68 in breast cancer cells inhibited cell proliferation by blocking the transition from G1 to S phase, and the Akt/GSK-3β signaling and FOXO/p21/p27 pathway appeared to be involved [11]. In early-stage cervical cancer, increased expression of SAM68 associated with lymph node metastasis apparently by promoting cellular motility and invasion, again through the Akt/ GSK-3β pathway [10]. In the current study, we explore the potential utility of SAM68 expression and localization in human bladder cancer, and report correlations with clinical outcomes, progression and prognosis.

## Methods

### Patients and tissue specimens

Patient consent and approval from the Sun Yat-sen University Cancer Center Institutional Review Board were obtained for the use of these clinical materials for research purposes. Ten pairs of MIBC tissue specimens and corresponding non-tumorous specimens were obtained from patients with bladder cancer who underwent radical cystectomy at the Cancer Center of the Sun Yat-sen University (Guangzhou, P. R. China). Eight paired of non-muscle invasive (NMIBC) and MIBC tissues from the same patient were obtained from TURBT and radical cystectomy, respectively. All excised tissues were obtained within 1 h after surgery and were immediately placed in liquid nitrogen until further analysis. Immunohistochemistry analyses were performed on 129 paraffin-embedded radical cystectomy samples, which were histologically diagnosed as MIBC at the Cancer Center, Sun Yat-sen University, between 2000 and 2008. Tumor-node-metastasis (TNM) staging was determined according to the 2010 American Joint Committee on Cancer TNM classification of bladder cancer [13]. The detail of patients’ information are summarized in Table 1. The median follow-up period for this cohort of patients was 32 months (range, 6-104 months). During the follow-up period, 35 patients had tumor recurrence.

**Table 1:** **Correlation between clinicopathological features and SAM68 expression in MIBC patients**

Characteristics		n	SAM68		*χ*^2^test*P*-value	n	SAM68			*χ*^2^test*P*-value
			Low	High			Nucleus	Cytoplasm	Nucleus and cytoplasm co-expression	
**Age (years)**	<60	63	32(50.8)	31(49.2)	0.292	62	25(40.3)	12(19.4)	25(40.3)	0.362
	≥60	66	27(40.9)	39 (59.1)		61	18(29.5)	11 (18.0)	32 (52.5)	
**Gender**	Male	116	55(47.4)	61 (52.6)	0.380	110	40(36.4)	21 (19.1)	49 (44.5)	0.497
	Female	13	4(30.8)	9 (69.2)		13	3(23.1)	2 (15.4)	8 (61.5)	
**pT stage**	T_2_	80	43(53.8)	37(46.3)	***0.028***	77	30(39.0)	18(23.4)	29(37.7)	***0.035***
	T_3~4_	49	16(32.7)	33(67.3)		46	13(28.3)	5(10.9)	28(60.9)	
**pN stage**	N^−^	111	55(49.5)	56(50.5)	***0.041***	105	39(37.1)	22(21.0)	44(41.9)	***0.050***
	N^+^	11	4(22.2)	14(77.8)		18	4(6.3)	1(5.6)	13(72.2)	
**Grade**	Low	32	12(37.5)	20(62.5)	0.312	31	11(35.5)	4(12.9)	16(51.6)	0.608
	High	97	47(48.5)	50(51.5)		92	32(34.8)	19(20.7)	41(44.6)	
**Smoking history**	Yes	68	29(42.6)	39(57.4)	0.483	65	23(35.4)	12(18.5)	30(46.2)	0.994
	No	61	30(49.2)	31(50.8)		58	20(34.5)	11(19.0)	27(46.6)	
**Gross hematuria**	Yes	102	43(42.2)	59(57.8)	0.132	97	32(33.0)	19(49.5)	46(47.4)	0.664
	No	27	16(59.3)	11(40.7)		26	11(42.3)	4(15.4)	11(42.3)	
**UIS**	Yes	18	8(44.4)	10(55.6)	1.000	17	5(29.4)	2(11.8)	10(58.8)	0.511
	No	111	51(45.9)	60(54.1)		106	38(35.8)	21(19.8)	47(44.3)	
**Tumor multiplicity**	Yes	70	34(48.6)	36(51.4)	0.595	66	18(27.3)	16(24.2)	32(48.5)	0.087
	No	59	25(42.4)	34(57.6)		57	25(43.9)	7(12.3)	25(43.9)	
**Adjuvant chemotherapy**	Yes	39	13(33.3)	26(66.7)	0.083	37	13(35.1)	4(10.8)	20(54.1)	0.295
	No	90	46(51.1)	44(48.9)		86	30(34.9)	19(22.1)	37(43.0)	
**Tumor recurrence**	Yes	35	9(25.7)	26(74.3)	***0.006***	32	6(18.8)	5(15.6)	21(65.6)	***0.031***
	No	94	50(53.2)	44(46.8)		91	37(40.7)	18(19.8)	36(39.6)	

MIBC: muscle invasive bladder cancer; UIS: urinary irritation symptoms.

### RNA extraction and quantitative PCR

Total RNA from tumor and adjacent non-tumorous tissues was extracted using the TRIzol reagent (Invitrogen) according to the manufacturer's instructions. Quantitative polymerase chain reaction (PCR) was performed according to standard methods as described previously [8]. PCR primers and probes were designed with the use of Primer Express Software v.2.0 (Applied Biosystems) as described previously [8].

### Immunohistochemistry

Immunohistochemistry (IHC) was performed to study altered SAM68 protein expression levels in 129 human MIBC tissues, as well as the ten pairs of MIBC tissue specimens and corresponding non-tumorous specimens, and eight paired of NMIBC and MIBC tissues. In brief, 4 μm-thick tissue sections were incubated with polyclonal rabbit antibody against SAM68 (1:200; Abgent) at 4°C overnight. Before incubation with the primary antibody, the sections were treated for antigen retrieval with ethylene diamine tetraacetic acid buffer followed by heating in a microwave oven. For negative controls, the rabbit anti-SAM68 antibody was restored with normal nonimmune serum. After washing, tissue pieces were treated with biotinylated anti-rabbit secondary antibody (Zymed), followed by further incubation with streptavidin -horseradish peroxidase complex (Zymed). Tissue sections were then immersed in 3,3′-diaminobenzidine and counterstained with 10% Mayer's hematoxylin, dehydrated, and mounted.

The degree of immunostaining of paraffin-embedded sections was reviewed and scored independently by two observers based on the proportion of positively-stained tumor cells and the intensity of staining. The method has been introduced in detail previously [8]. The staining index was calculated as the product of the staining intensity score and the proportion of positive tumor cells. Using this method of assessment, we evaluated SAM68 expression in MIBC tissues by determining the staining index, with scores of 0, 1, 2, 3, 4, 6, 8, 9, and 12. SAM68 threshold values were chosen on the basis of a measure of heterogeneity with the log-rank test statistical analysis with respect to recurrence-free survival. An optimal threshold value was identified: a staining index score >6 was considered to show high SAM68 expression, whereas a staining index score <4 was considered to represent low SAM68 expression.

### Statistical analysis

The relationship between SAM68 expression and clinicopathological characteristics was analyzed by the chi-square and Fisher’s exact tests. Survival curves between subgroups that were divided according to SAM68 expression levels and localizations were drawn with the use of the Kaplan-Meier method, and significant differences among subgroups were compared by the log-rank test. Survival data were evaluated using univariate and multivariable Cox regression analyses. In all cases, P < 0.05 was considered to be statistically significant. All statistical analyses were conducted using the SPSS v.13.0 statistical software package (SPSS, Chicago, IL, USA).

## Results

### The expression of SAM68 in paired of MIBC tissues and adjacent normal bladder urothelium

**Figure 1:**
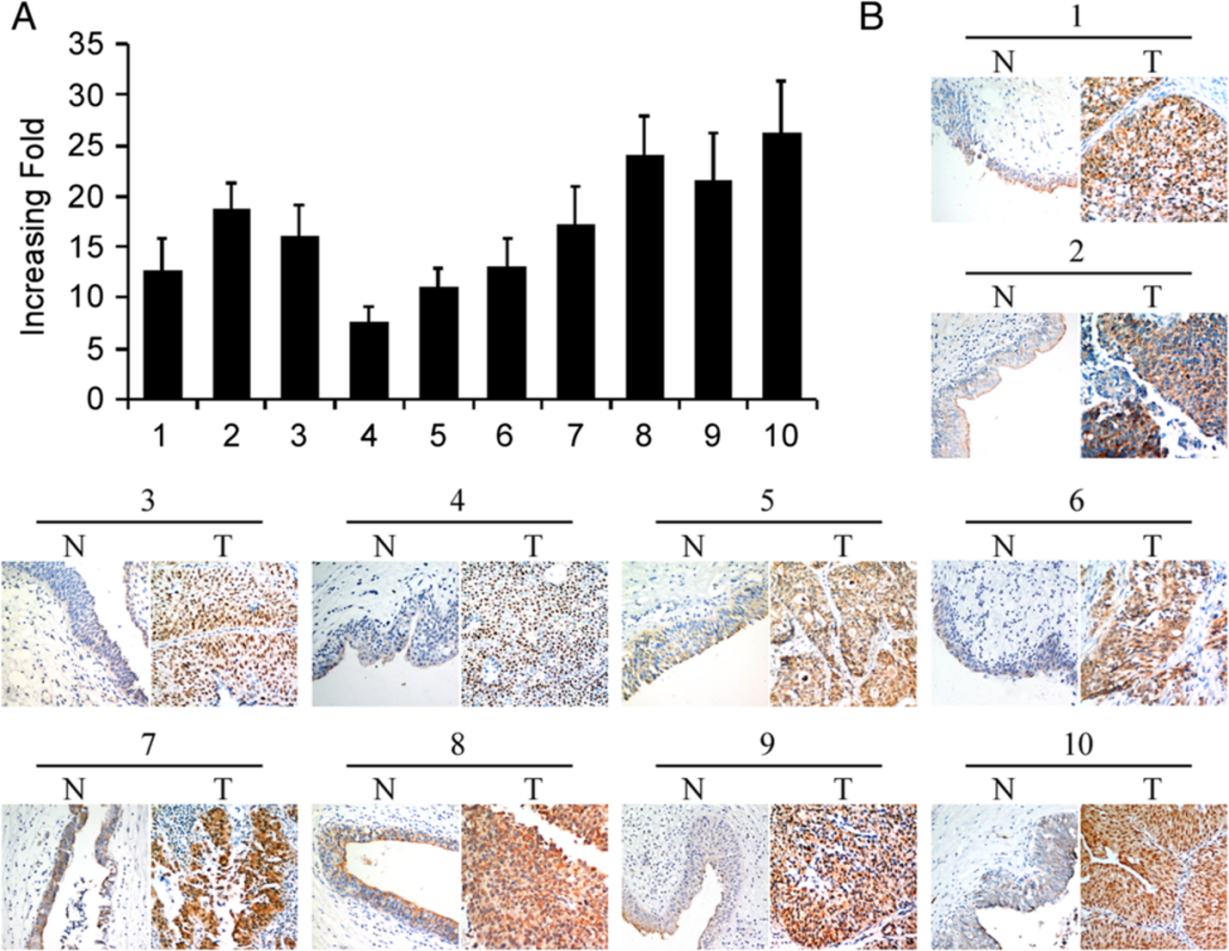
Expression of SAM68 mRNA and protein in paired muscle invasive bladder cancer tissues and adjacent non-carcinoma tissues. **(A)** Quantitative PCR showed the mRNA level of SAM68 was much higher in bladder cancer tissues compared with adjacent non-carcinoma tissues. Expression levels were normalized for *GAPDH*. Error bars represent the standard deviation (SD) calculated from three parallel experiments. **(B)** Immunohistochemistry analysis was performed to detect the SAM68 protein level in tumor (T) and non-carcinoma tissues (N) from the same patient.

To determine the expression of SAM68 in MIBC tissues, quantitative PCR analyses were conducted on ten matched pairs of bladder cancer tissues and adjacent, nontumorous bladder urothelium from the same patient. As shown in Figure 1, all the ten bladder cancer tissues showed up-regulated SAM68 mRNA expression, when compared with their adjacent bladder urothelium. IHC analysis further confirmed this result in the translation level (Figure 1B). These data suggests that SAM68 expression was elevated in human MIBC.

### The expression of SAM68 in paired of NMIBC and MIBC tissues

**Figure 2:**
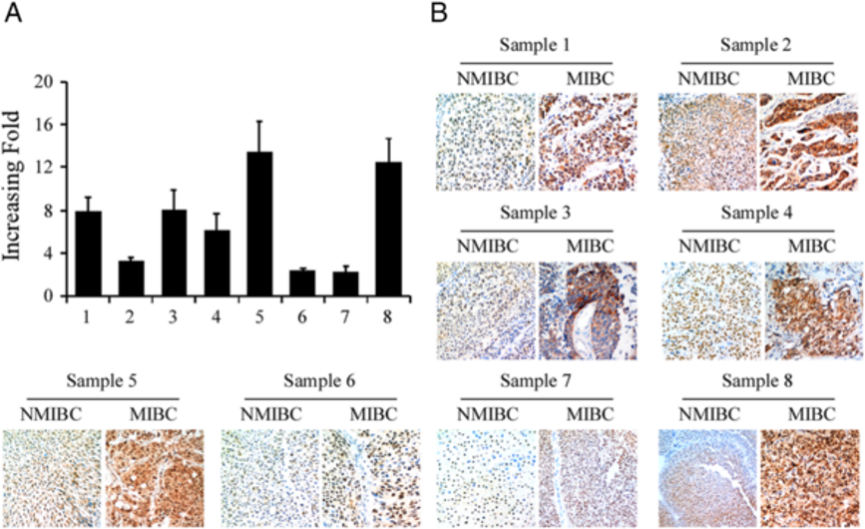
Expression of SAM68 protein was increased when bladder tumor developed from non-muscle invasive to muscle invasive. **(A)** Increased fold of SAM68 mRNA for muscle invasive tumor comparing to non-muscle invasive tumor from the same patient. **(B)** Immunohistochemistry study confirmed the expression of SAM68, in the protein level, was much higher in muscle invasive tumor samples than non-muscle invasive tumor from the same patient. For each pair of sample, the left panel represents non-muscle invasive bladder cancer (NMIBC) sample obtained from transurethral resection of bladder tumor and the right panel represents muscle invasive bladder cancer (MIBC) sample gained from radical cystectomy.

To explore the role of SAM68 in bladder cancer progression, we analyzed eight MIBC patients with a history of NMIBC, who underwent radical cystectomy for recurrent tumor infiltrated into or beyond muscularis propria. Paired of NMIBC and MIBC were obtained from TURBT and radical cystectomy, respectively, of the same patient. The results indicated that SAM68 was markedly up-regulated at both transcriptional and translational levels in MIBC tissues compared with NMIBC tissues by quantitative PCR and IHC (Figure 2A and B). These findings suggested that bladder cancer developing from NMIBC to MIBC may be accompanied with SAM68 expression level ascending.

### The expression of SAM68 in 129 MIBC tissues from radical cystectomy

**Figure 3:**
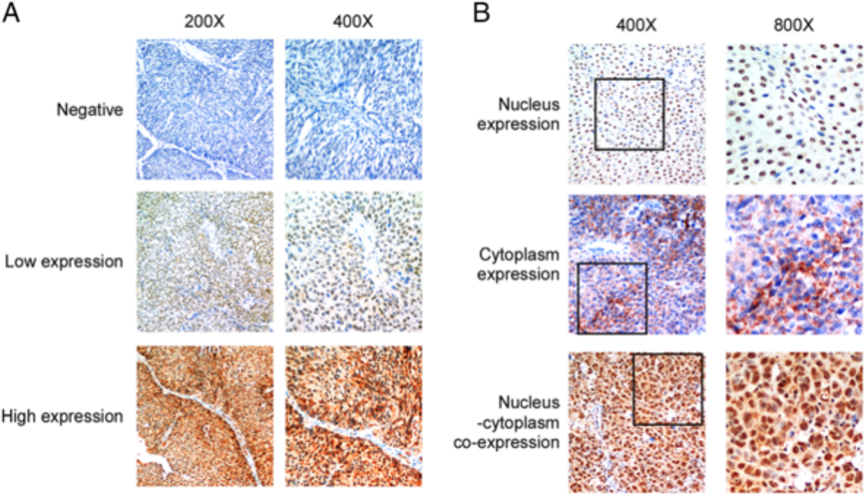
Typical images showed the expression level and localization of SAM68 protein as examined by immunohistochemistry (IHC). **(A)** Series showed the negative, low level and high level expression of SAM68 protein. Left panel: 200×; right panel: 400×. **(B)** Three types of SAM68 expression according to the intracellular localization: nucleus expression, cytoplasm expression and nucleus-cytoplasm co-expression. Left panel: 400×; right panel: 800 × .

To study the clinicopathological significance of SAM68 in MIBC, we examined the SAM68 protein expression levels in 129 paraffin-embedded, archived MIBC, from radical cystectomy, with long term follow-up data. The IHC results are summarized in Table 1. SAM68 protein was detected in 123 out of 129 (95.3%) human MIBC samples, in which 70(54.3%) showed high expression of SAM68 protein and 59(45.7%) showed low expression of SAM68 protein (Figure 3A). For the localization of SAM68 protein, there were three types of expression pattern, including nucleus expression, cytoplasm expression and nucleus-cytoplasm co-expression (Figure 3B). 43 cases (35.0%) had SAM68 expression in nucleus, 23 cases (17.8%) in cytoplasm and 57 cases (46.3%) had SAM68 expressed in both nucleus and cytoplasm.

### High expression and nucleus-cytoplasm co-expression of SAM68 were associated with clinical features and recurrence-free survival

**Figure 4:**
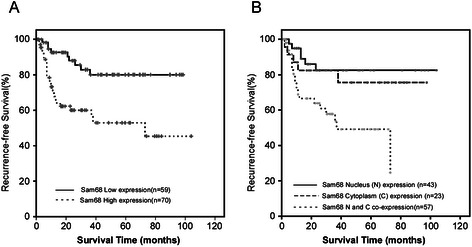
Kaplan-Meier curves with log-rank test comparing recurrent free survival (RFS) in different subgroups **(A)** Bladder cancer patients with low SAM68 expression (bold line) had a cumulative 5-year RFS rate of 80.0%, compared to 52.9% for patients with high SAM68 expression (dotted line; p = 0.001). **(B)** Comparison of RFS curves between groups with different SAM68 intracellular localization. The RFS curves were similar between SAM68 nucleus expression and cytoplasm expression groups (p = 0.539). However, the RFS was much worse in the nucleus-cytoplasm co-expression group, no matter compared with nucleus expression group (p = 0.002) or cytoplasm expression group (p = 0.037).

To determine the association of SAM68 expression and clinical features and recurrence-free survival of MIBC, *χ*^2^ test and survival analysis with long-rank test were performed. As shown in Table 1, high expression and nucleus-cytoplasm co-expression of SAM68 were correlated with the T stage (p = 0.028, and p = 0.035), N stage (p = 0.041, and p = 0.050) and recurrence status (p = 0.006, and p = 0.031). The Kaplan-Meier survival analysis indicated that patients who had high SAM68 expression levels had worse recurrence-free survival than those with low SAM68 expression. The low SAM68 expression group had a cumulative 5-year recurrence- free survival rate of 80.0% (95% confidence interval [CI], 0.680-0.919), whereas the high SAM68 expression group only had 52.9% (95% CI, 0.384-0.674) (Figure 4A). Patients with SAM68 nucleus-cytoplasm co-expression had lower 5-year recurrence- free survival rate (49.2%) than those with SAM68 expressed in nucleus (82.5%) or cytoplasm (75.5%). However, the difference of recurrence-free survival curve between SAM68 nucleus group and cytoplasm group is not significant (Figure 4B). In addition, the multivariable analysis revealed that SAM68 expression level, nucleus-cytoplasm co-expression, accompanied with T stage, and N stage were independent prognostic factors for the recurrence-free survival rate of MIBC patient (Table 2). These results indicated that SAM68 has potential as a valuable prognostic marker for MIBC after radical cystectomy.

**Table 2:** **Univariate and Multivariate analyses of various prognostic parameters in patients with MIBC: Cox-regression analysis**

	Univariate analysis			Multivariate analysis		
	HR	95% CI	p Value	HR	95% CI	p Value
pT stage (T3 ~ 4 vs. T2)	4.445	2.221 ~ 8.896	***<0.001***	2.308	1.047 ~ 5.089	***0.038***
pN stage (N^+^ vs. N^−^)	4.944	2.313 ~ 10.780	***<0.001***	2.879	1.222 ~ 6.872	***0.016***
SAM68 level (High vs. low)	3.366	1.574 ~ 7.201	***0.002***	2.365	1.055 ~ 5.298	***0.037***
SAM68 Localization*	2.003	1.267 ~ 3.166	***0.003***	1.749	1.106 ~ 2.767	***0.017***

MIBC: muscle invasive bladder cancer; HR: hazard ratio; CI, confidence interval.

*nucleus-cytoplasm co-expression vs. expression in nucleus vs. expression in cytoplasm.

## Discussion

MIBC is typically managed with perioperative chemotherapy and radical cystectomy with extended pelvic lymph node dissection [14], yet almost 50% of such patients will eventually succumb to disease progression [14,15]. In addition, even for survivors, quality of life is often compromised due to the urinary diversion, erectile dysfunction, or other functional concerns [16,17]. Availability of prognostic biomarkers would greatly improve our management of patients with bladder cancer by facilitating better patient selection and individualized risk stratification. Previous efforts have focused primarily on cell cycle regulators, but the clinical utility of currently available markers remains limited. For instance, Margulis and colleagues [18] studied Ki-67, a marker of proliferation, after radical cystectomy and reported an independent association of high Ki-67 labeling index with disease recurrence and cancer-specific mortality. Another meta-analysis [19] pooled the results of 16 studies which investigated the role of increased p53 expression for predicting the prognosis of bladder cancer, reporting an overall hazard ratio of 1.43 for predicting mortality. However, due to the complicated biological behavior of bladder cancer, none of these markers have been widely used in clinical practice. Hence, there is still a great need to identify novel predictive factors for bladder cancer.

In the current study, we evaluate SAM68 as a potential prognostic marker for MIBC, and report for the first time clinical and pathologic correlates for this malignancy. Using immunohistochemistry, we found that MIBC patients with high expression or nucleus-cytoplasm co-expression of SAM68 had lower recurrence-free survival at 5 years. Multivariable analysis revealed that SAM68 expression level and nucleus-cytoplasm co-expression were independent prognostic factors for recurrence-free survival, adding additional prognostic power above that provided by T-stage and N-stage, which are the well-established prognostic factors for this malignancy. Our data suggests that SAM68 may allow for refined risk stratification after radical cystectomy that could prove to be of clinical utility. In particular, patients with high expression or nucleus-cytoplasm co-expression of SAM68 may need adjuvant therapy and more intensive surveillance. If identified on biopsy, SAM68 could potentially influence decisions about neoadjuvant chemotherapy or even less intensive alternative strategies, such as chemo-radiation, although further studies will be required. In addition, we found that bladder cancer developing from NMIBC to MIBC may be associated with increased SAM68 expression, which if validated in independent and larger cohorts, might suggest a potential target for therapeutics.

Our findings are consistent with recent data about SAM68 as a tumor-promoter in other cancers [8,10,11,20], although the mechanism of action is not well defined. Consistent with this, we found that the expression level of SAM68 was much higher in MIBC when compared to adjacent normal bladder urothelium or NMIBC tissue from the same patient. Furthermore high expression of SAM68 associated with worse clinicopathological parameters and prognosis. These findings supported SAM68 as a potential tumor-promoter in bladder cancer, although a surrogate effect cannot be excluded, and the biological basis for this will require further investigation. In other cancers, the function and mechanism of action of SAM68 in cancer progression has been revealed to some degree. In LNCaP prostate cancer cells, reduced expression of SAM68 altered the expression of an important subset of genes involved in proliferation and apoptosis, including Bcl2L1, Clusterin, cdk2, cdk3, p16INK4, cyclin D1, Par-4, EGF and IGF-1 [21]. In breast cancer cells, silencing SAM68 resulted in anti-proliferative effects that appeared to be due to up-regulation of p21 and p27 and attenuation of Akt/GSK-3β signaling [11]. Further studies will be required in bladder cancer to determine if similar or alternate mechanisms of action are operative.

Another interesting finding was that almost 50% of MIBC cases demonstrated co-expression of SAM68 in both the nucleus and cytoplasm, which associated with advanced T and N stage and compromised recurrence-free survival, and co-expression proved to be an independent prognostic factor on multivariable analysis. Although SAM68 was initially reported to be predominantly localized to the nucleus [22,23], cytoplasmic localization has also been observed in several human cancers and has shown clinical significance, particularly for breast cancer and early-stage cervical cancer [10,11]. Although the function and mechanism of SAM68 accumulation within the cytoplasm remains unknown, several hypotheses have been proposed. Some studies have suggested that cytoplasmic SAM68 may associate with polysomal mRNAs and enhance the translational efficiency of eEF1, an elongation factor which has been implicated in cellular transformation [21,24-26]. It is particularly noteworthy in our study that the prognosis was much worse in the nucleus and cytoplasm co-expression group compared with expression isolated to either the nucleus or cytoplasm, while the prognosis of the latter two groups appeared to be similar. A possible explanation for this phenomenon is that SAM68 causes poor prognosis of bladder cancer by pulling two triggers in both the nucleus and cytoplasm simultaneously, which may act in a synergistic manner, although further research will be required to study such potential mechanisms.

Although our study enrolled a large cohort of MIBC patients, there are still some limitations. Firstly, this was a retrospective study, and some bias is therefore inevitable. Secondly, the mechanism and function of the accumulation of SAM68 in the cytoplasm were not investigated in this study. Thirdly, the role of high expression of SAM68 in bladder cancer cell progression has not been illuminated. Finally, independent validation with larger cohorts of patients will be required to more definitively explore the potential value of SAM68 as a biomarker for bladder cancer.

## Conclusions

In summary, our study suggests that SAM68 expression is elevated in MIBC compared with normal urothelium and NMIBC and may have potential as a prognostic marker for MIBC. However, large scale prospective study will be required to validate our findings, and further studies regarding mechanisms of action are also indicated.

## Abbreviations

MIBC: Muscle invasive bladder cancer
NMIBC: Non-muscle invasive bladder cancer
TURBT: Transurethral resection of bladder tumor
TNM: Tumor-node-metastasis
PCR: Polymerase chain reaction
IHC: Immunohistochemistry
